# *Lactococcus garvieae* Endocarditis in a Prosthetic Aortic Valve: A Case Report and Literature Review

**DOI:** 10.1177/2324709619832052

**Published:** 2019-04-22

**Authors:** Romina Maria Rösch, Katja Buschmann, Lena Brendel, Thomas Schwanz, Christian-Friedrich Vahl

**Affiliations:** 1 University Medical Center Mainz, Mainz, Germany

**Keywords:** *Lactococcus garvieae* infections, infective endocarditis, heart valve prosthesis

## Abstract

*Background.* Lactococcus garvieae (LG) is a gram-positive coccus known to be a major pathogen in aqua farming, which is responsible for severe outbreaks. Its incidence in humans is extremely rare. Prior to 1985, all bacteria in the genus Lactococcus were included in the Streptococcus genus. The first human infection was documented in 1991, and since then, the relevance and clinical significance in humans has increased. *Case Description.* We present the clinical course of an LG endocarditis in a 78-year-old man who had a history of exertional dyspnea. The patient’s blood tests showed increased inflammation values, and a transesophageal ultrasound (TEE) showed a stenosis of the prosthetic aortic valve. Blood cultures were positive for LG, leading to a diagnosis of infective endocarditis. After 6 weeks of intravenous antibiotics and a prosthetic aortic valve replacement, the patient made a good recovery. Review of the Literature. After the first documented case in 1991 to 2018, 25 cases of LG endocarditis have been described in PubMed and MEDLINE. We reviewed all reported cases of LG endocarditis, commenting on predisposing risk factors, the course and outcome of the disease. *Conclusion.* LG endocarditis is a rare disease. Consumption of raw fish, abnormalities of the digestive tract, immune deficiency, and underlying cardiac conditions appear to be risk factors for an infective endocarditis due to LG. Improved determination techniques are likely to lead to a better and faster identification of the bacterium. This identification allows a faster and individualized therapy, which in turn affects the outcome.

## Introduction

*Lactococcus garvieae* is a catalase-negative, facultative anaerobic, nonhemolytic, gram-positive chain cocci^1^ present throughout the world. It is also known as *Enterococcus seriolicida*.^2^

*Lactococcus garvieae* was first described in 1983.^3^ Since then, it has been described frequently in the literature as a highly opportunistic pathogen in aqua farming, and outbreaks of *L garvieae* infections in fish colonies significantly affect production.^4^

*Lactococcus garvieae* proliferation peaks during summer, when the water temperature rises, and this also coincides with a rise in the number of *L garvieae* infections in fish.^4^

The first case of human infection with *L garvieae* was reported in 1991.^5^ Since then, various types of infections have been described, including lumbar osteomyelitis, meningitis, hepatic abscess, and infective endocarditis (IE).^6^

The relevance of *L garvieae* has increased in recent decades, as has its clinical significance in humans.^7^ However, meager information is currently available about the process of transmission to humans. We assume the infection follows the consumption of raw fish, especially in patients with digestive disorders, which was previously described in other cases of *L garvieae* IE.^8^ More recently, studies carried out on dairy products obtained from raw milk suggest another possible ecological niche as an origin of *L garvieae*.^2^

It is difficult to distinguish *L garvieae* from enterococci,^9^ and misidentifications of *L garvieae* as Enterococcus species are common in small clinical microbiology laboratories.^7^ Laboratories in tertiary hospitals are more easily able to identify *L garvieae* species.

Different molecular methods, such as API strips, BD Phoenix, the VITEK system, and MicroScan, have been examined to identify L garvieae from clinical specimens.^1,10-14^ *L garvieae* can also be obtained by specific polymerase chain reaction (PCR) amplification.^15^ True identification is now possible due to the use of mass spectrometry, especially matrix-assisted laser desorption ionization time-of-flight (MALDI-TOF). MALDI-TOF mass spectrometry has emerged in recent years as an alternative to conventional identification techniques for the accurate, rapid, and inexpensive identification of a broad spectrum of pathogenic bacteria in clinical microbiology laboratories.^16^

The treatment is not yet standardized because no test has been established to determine the exact criteria of susceptibility screening.^17^ Due to the similarity between *L garvieae* and streptococcal infections, treatment plans are often the same or comparable. These treatments usually require high doses of β-lactams, including ampicillin, amoxicillin, or ceftriaxone, administered alone or in combination with aminoglycosides such as netilmicin, tobramycin, or gentamicin. In cases where no β-lactams are possible, aminoglycosides could be paired with glycopeptides such as vancomycin or teicoplanin.^18,19^ Other combinations, with the risk of more side effects, contain the application of ceftriaxone/levofloxacin or vancomycin/gentamicin.^1,20^

The low prevalence of *L garvieae* infections in humans, despite physicians increasing awareness of the clinical relevance of *L garvieae* as a human pathogen, can be explained by incorrect interpretation of other streptococcal species and, in the past, by a lack of sufficient laboratory equipment. Only 25 cases of IE due to *L garvieae* have been described since 1991,^5^ when it first was identified as the cause of an IE. We will describe *L garvieae* IE in a patient who has an aortic prosthetic valve replacement.

## Case Presentation

We present a case of a 78-year-old male with extensive cardiac history, including paroxysmal atrial fibrillation, essential hypertension, chronical renal dysfunction (III-IV, conservative therapy), stenosis of the right external carotid artery, and stenting of the right coronary artery. The patient underwent cardiac surgery including aortic valve replacement (Medtronic Hancock II, 23 mm) and coronary artery bypass grafting (LIMA-LAD, LIMA-PLA T-Graft) 1½ years prior this presentation.

He was admitted to the hospital with a 5-day history of exertional dyspnea NYHA III (New York Heart Association Class III) and expectoration. On admission, he had a tympanic temperature of 38.5°C, a heart rate of 71 beats/min, blood pressure of 148/68 mm Hg, and oxygen saturation of 97% of room air. During his physical examination, there was an aortic systolic murmur (4/6) on auscultation. His medications included mucolytic, aspirin, β-blocker, statin, diuretic, pantoprazole, and rivaroxaban. He was admitted for progressive heart failure with a fever of 38.5°C and chills.

### Laboratory Findings

Admission laboratory results were significant for a white blood cell count of 20.1 × 10^9^/L, a C-reactive protein level of 209 mg/L, and an interleukin-6 level of 24.93 pg/mL (<6.4 pg/mL). Four blood cultures were drawn, and it was then started to treat the patient with empirical antibiotics (vancomycin, gentamicin, and rifampicin) due to concern for prosthetic valve IE.

**Figure 1.**
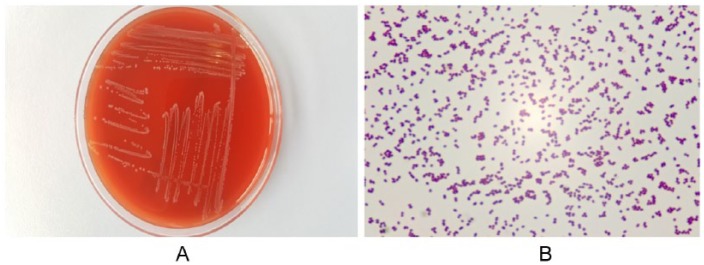
(A) Growth of colonies of *Lactococcus garvieae* on a blood agar plate after 48 hours of incubation in aerobic conditions. (B) Gram stain of *Lactococcus garvieae* with clusters and short chains of gram-positive cocci.

Three out of 4 blood cultures were positive for gram-positive chain cocci, and 4 more blood cultures were drawn. After 48 hours, *L garvieae* had grown in all 3 positive blood cultures. *L garvieae* was also found in the repeated blood cultures (Figure 1).

*Lactococcus garvieae* was susceptible to ampicillin, vancomycin, cefotaxime, ceftriaxone, penicillin, and gentamicin (high level). It was resistant to clindamycin, which has previously been described in other cases. Empiric antibiotic treatment was completed for 6 weeks with 4 million units of penicillin given every 4 hours. During the first 2 weeks, gentamicin was also given, initially 320 mg given every 24 hours, then later reduced to 240 mg every 24 hours.

A transesophageal ultrasound (TEE) was performed and we obtained the results described below. Left atrial appendage without detection of thrombotic material with good flow velocity (>0.5 m/s). Atrial septum without detection of atrial septal defect or patent foramen ovale. Aortic valve replacement (Hancock II, 23 mm) morphologically with degenerative stenosis (V_max_ 5.42 m/s, P_max_ 117 mm Hg, and P_mean_ 71 mm Hg) and visually impaired pocket valve separation (AVAI [indexed aortic valve area] = 0.6 cm^2^) and pedunculated echogenic material of 10-mm diameter on the prosthetic aortic valve, no paravalvular leak. No vegetation was seen on pacemaker leads. Mitral valve with degenerative change emphasizes posterior mitral leaflet/anulus with central mitral valve insufficiency I°/II°. Tricuspid valve was inconspicuous as far as visible. No tricuspid valve insufficiency. Aorta with evidence of arteriosclerotic changes. An abdominal ultrasound showed shrunken kidneys and an enlarged spleen.

### Operational Approach and Findings

Five days after admission, the patient underwent surgery for the repeated procedure. After careful preparation, the heart valve was removed, and the vitreous deposits were extracted. Intraoperative findings showed thrombotic/fibrinous flat deposits, which were attached to the valve leaflets. After the explanation of the prosthetic aortic valve and the removal of the infected tissue, an aorta ascendens enlargement plastic (Manugian) was needed and performed. A Medtronic Hancock II Porcine, 21 mm, was inserted into the aortic valve position. The intraoperative TEE showed no evidence of paravalvular leaks or prosthetic heart valve insufficiency. The following intensive care stay was uneventful, as well as the rest of the patient’s hospitalization.

### Microbiological Findings and Genome Detection of *Lactococcus garvieae*

Microscopic examination of the prosthetic aortic valve showed sclerotic tissue, and microbiological testing detected *L garvieae* bacterial as well as inflammatory cells, in the form of granulocytes. Microbiological testing of the blood culture detected *L garvieae* by using MALDI-TOF mass spectrometry. The final diagnosis was IE caused by *L garvieae*.

The patient was discharged on postoperative day 8, after 11 days of intravenous antibiotics, which were continued during the patient’s post-hospitalization rehabilitation. The patient underwent a total course of 6 weeks of penicillin and 2 weeks of gentamicin.

**Figure 2.**
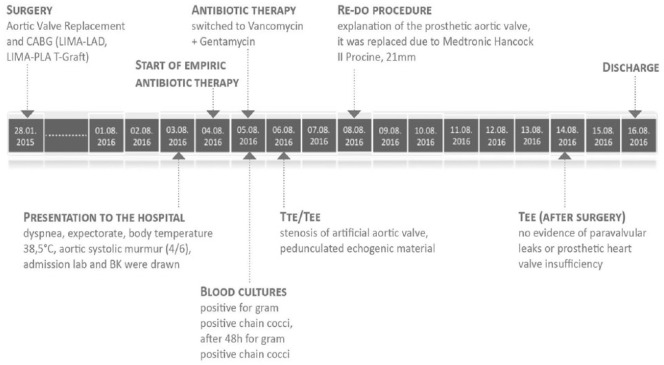
Timeline of patient’s medical history.

During the 6-month follow-up period, the patient was free of *L garvieae* IE (Figure 2).

## Discussion

Infective endocarditis is a common disease, especially in patients with heart valve dysfunction or after a prosthetic valve replacement. IE is most commonly caused by *Streptococcus viridans* and staphylococci group.

*Lactococcus garvieae* is an unusual human pathogen and seems to behave like an opportunistic agent with low virulence.^21^ Prior to the case we describe above, only 25 cases (Table 1) of IE due to *L garvieae* have been described since its first description in 1991. *L garvieae* has been related to a variety of different opportunistic infections.^10,11,14,20,22-24^

**Table 1.** Table of LG endocarditis cases described in the literature (incidence, demographics, type, risk factors, antibiotics, surgery, outcome).

Reference	Acquired Infection Location	Sex/Age	Type	Risk Factors	Antibiotics	Surgery	Outcome
Navas et al^13^	USA	Male/64	NAV		VAN	Yes	Survival
Ortiz et al^25^	Spain	Female/70	NMV		AMX + GEN	Yes	Survival
Ortiz et al^25^	Spain	Female/77	NMV/NAV	GD	AMX + GEN	No	Death
Backes et al^26^	Holland	Female/68	NAV	GD		No	Survival
Zuily et al^8^	France	Female/64	PMV	RF/GD	AMX + GEN	No	Survival
Heras et al^27^	Spain	Male/68	NMV		GEN + AMP + CTX	Yes	Death
Watanabe et al^17^	Japan	Female/55	NMV		PEN + GEN, CTX + GEN	No	Survival
Li et al^12^	China	Male/41	NMV		PEN + GEN	Yes	Survival
Clavero et al^28^	Chile	Female/72	NMV	RF	AMP + CLX, VAN + GEN	No	Death
Rasmussen et al^29^	Sweden	Male/81	PAV/NMV	GD	PEN + TOB	No	Survival
Bazemore et al^30^	USA	Male/45	PAV	GD	VAN + Pip/Taz, CTX + GEN	Yes	Survival
James et al^22^	UK	Female/56	PAV		VAN	No	Survival
Tsur et al^31^	Israel	Male/76	PAV	GD	CTX + GEN	No	Survival
Russo et al^1^	Italy	Male/63	PAV/NMV	GD	VAN + GEN, AMP + GEN	No	Survival
Hirakawa et al^32^	Brazil	Female/58	PMV	RF	VAN	No	Survival
Suh et al^33^	Korea	Female/75	PMV	RF	CTX + GEN + RI, TEI + CTX	Yes	Survival
Wilbring et al^34^	Germany	Male/55	PTV		VAN + GEN, AMX + LEV	No	Survival
Landeloos et al^35^	Belgium	Female/82	PMV	GD	AMX, PEN + GEN	No	Survival
Lim and Jenkins^36^	UK	Male/57	NMV	GD	AMX + GEN	Yes	Survival
Wang et al^37^	China	Male/72	NMV	RF/GD	PEN + GEN	No	Survival
Fleming et al^18^	Korea	Male/68	PAV/NMV	RF/GD	VAN	No	Death
Fihman et al^21^	France	Female/86	PAV	GD	AMX + GEN	No	Survival
Vinh et al^6^	Canada	Male/80	NAV	GD	AMP	Yes	Survival
Yiu et al^38^	China	Male/67	NMV		AMP	Yes	Survival
Fefer et al^39^	USA	Female/84	NMV/PAV		CTX	Yes	Death

Abbreviations: NAV, native aortic valve; VAN, vancomycin; NMV, native mitral valve; AMX, amoxicillin; GEN, gentamicin; GD, gastrointestinal disorder; PMV, prosthetic mitral valve; RF, raw fish; AMP, ampicillin; CTX, ceftriaxone; CLX, cloxacillin; PAV, prosthetic aortic valve; PEN, penicillin; TOB, tobramycin; Pip/Taz, piperacillin/tazobactam; RI, rifampicin; TEI, teicoplanin; PTV, prosthetic tricuspid valve; LEV, levofloxacin.^1,6,8,12,13,17,18,21,22,25-39^

Including our case, a total of 26 cases of IE caused by *L garvieae* have been reported in the literature. The infection has occurred in the following areas: 15.4% in North America, 7.7% in Latin America, 23% in Asia, and 50% in Europe. One infection (3.9%) occurred in the Middle East (Israel). Fourteen patients were men (53.8%), and 12 were women (46.2%). The mean age was 68 years (67.8 years), and 18 patients were 60 years of age or older (69.2%).

Eleven patients underwent cardiac surgery, and 4 of the 11 were repeated procedures. Seventeen cases (65.4%) involved a native valve. Prior the case we describe above, the previous 25 cases included 5 patients (20%) with multivalve IE, for a total of 31 affected valves. Among these were 4 native aortic valves (12.9%), 13 native mitral valves (41.9%), 8 prosthetic aortic valves (25.8%), prosthetic mitral valves, and 1 prosthetic tricuspid valve (3.2%).

Of these 25 cases, 5 patients (19.2%) died due to multi-organ failure, heart failure, or cerebral hemorrhage.^18,25,27,28,39^

The exact mechanism leading to transmission to humans is not yet established. Previous case reports assume that *L garvieae* infection occurred due to a barrier gap in the digestive tract (portal of entry) while consuming contaminated food, especially raw fish or fermented milk products. This fits with the observation that most of the *L garvieae* infections are described during summer and in patients who have a history of consuming raw fish.^4^ The number of *L garvieae* infections in fish also peak in the summer months (June, July, and August) when the water temperature is warmest.

This study shows that out of the 26 cases, 6 cases (23%) had a history of prior raw fish consumption. Gastrointestinal disorders (potentially leading to barrier gaps) were present in 13 cases (53.8%; 6 colonic polyps, 3 colonic/rectal diverticulosis, 1 gastric ulcer, 1 duodenal ulcer, 2 colorectal cancer).

Our patient was immunocompromised and had a postoperative ileus 1½ years ago and colorectal cancer, which could have facilitated the infection, together with the predisposing factor of a biological prosthetic valve. However, he denied eating raw fish in the last couple of weeks prior to admission to the hospital.

Even with only 26 cases described in the world, an infection due to *L garvieae* requires specific care and therapy. IE may be higher than reported in the literature and might be explained due to the fact that it is often misdiagnosed. *Lactococcus* is difficult to distinguish from *Enterococcus*,^9^ and some microbiological laboratories do not have the optimal equipment and capabilities to differentiate this bacterium. Although *L garvieae* is considered opportunistic, there is more need for research in this area.

In conclusion, the best way to identify *L garvieae* is through 2 or 3 sets of blood cultures and, in case of surgery, by analyzing a sample of the removed valve. With this material, it is possible to distinguish *L garvieae* by MALDI-TOF mass spectrometry and the 16s rRNA gene PCR, in addition to finding a susceptibility profile. In order to determine the final diagnosis of IE, the clinical symptoms, the microbiological findings, and the imaging procedures (transthoracic echocardiogram, TEE) must be evaluated using the Duke criteria.

The presented case fulfills the modified Dukes criteria of IE^40^ with 1 major criterion (TEE: vegetation on the prosthetic valve) and 3 minor criteria (fever >38°C, prosthetic heart valve, positive blood cultures—not listed as typical pathogen).

All 26 patients were treated with antibiotic therapy, mainly β-lactam antibiotics. The identified organism in this case appeared in the antimicrobial susceptibility test (Etest method) and showed a susceptibility to ampicillin, vancomycin, cefotaxime, ceftriaxone, penicillin, and gentamicin (high level). It was resistant to clindamycin, similar to other described cases of *L garvieae* (Table 2).

**Table 2.** Microbiological Findings: Susceptibility Testing of *Lactococcus garvieae*.

Antibiotic	MIC (mg/L)	MIC Interpretation^a^
Penicillin	0.25	S
Ampicillin	0.5	S
Ceftriaxone	0.25	S
Cefotaxime	0.25	S
Gentamicin	6	S
Clindamycin	>1.0	R
Vancomycin	1.0	S

Abbreviations: MIC, minimal inhibitory concentration; S, sensitive; R, resistant.

a Because of the lack of standardized susceptibility testing, the MIC interpretation is of orienting character only.

The treatment for *L garvieae* IE is not yet standardized because the exact criteria of the susceptibility test have not been established. In the case described above, the patient received a 6-week course of intravenous penicillin, 4 million units every 4 hours, and a 2-week course of gentamicin, 320 mg every 24 hours, later reduced to 240 mg every 24 hours.

## Conclusion

*Lactococcus garvieae* endocarditis is a rare disease in humans. Including our case, a total of 26 cases have been reported in the world.^1^

Prior to 1985, *Lactococcus* bacteria were included in the *Streptococcus* genus. The first human infection with *L garvieae* was documented in 1991, and since then, the relevance and clinical significance in humans has increased. Improved determination techniques, such as MALDI-TOF mass spectrometry and the 16s rRNA gene PCR, are likely to lead to a better and faster identification of the bacterium.

As observed in other cases of *L garvieae* endocarditis, consumption of raw fish, abnormalities in the digestive tract, and underlining cardiac conditions (prosthetic valve) seem to be risk factors for an IE due to *L garvieae*. This bacterium seems to mainly affect elderly and immunocompromised individuals with underlying cardiac conditions or those with anatomical or physiological alterations of their gastrointestinal tract. However, there have also been infections described in young and healthy people.

On the basis of existing literature of *L garvieae* endocarditis cases, it is assumed that *L garvieae* exposure can lead to infections in immunosuppressed humans via raw fish consumption and/or with underlying gastrointestinal disorders.

In the future, consideration should be given to examining patients with *L garvieae* endocarditis for changes in their gastrointestinal tract as a potential port of entry for infection.
